# Possible involvement of PS-PLA1 and lysophosphatidylserine receptor (LPS1) in hepatocellular carcinoma

**DOI:** 10.1038/s41598-020-59590-3

**Published:** 2020-02-14

**Authors:** Baasanjav Uranbileg, Makoto Kurano, Masaya Sato, Hitoshi Ikeda, Takeaki Ishizawa, Kiyoshi Hasegawa, Norihiro Kokudo, Yutaka Yatomi

**Affiliations:** 1 Department of Clinical Laboratory Medicine, The University of Tokyo, Tokyo, Japan; 2 Hepato-Biliary-Pancreatic Surgery Division, Department of Surgery, The University of Tokyo, Tokyo, Japan; 3 Japan Agency for Medical Research and Development, Core Research for Evolutional Science and Technology (AMED-CREST), Tokyo, Japan

**Keywords:** Phospholipids, Hepatocellular carcinoma

## Abstract

Lysophosphatidylserine (LysoPS) is a lysophospholipid, its generating enzyme, phosphatidylserine-specific phospholipase A1 (PS-PLA1), reportedly plays roles in stomach and colon cancers. Here, we examined the potential roles of LysoPS in hepatocellular carcinoma (HCC). The ninety-seven HCC patients who underwent surgical treatment were enrolled in this study and approved by the institutional review board. Among LysoPS-related enzymes and receptors, increased PS-PLA1 or LysoPS receptor 1 (LPS1) mRNA was observed in HCC tissues compared to non-HCC tissues. PS-PLA1 mRNA in HCC was associated with no clinical parameters, while LPS1 mRNA in HCC was correlated inversely with tumor differentiation. Furthermore, higher serum PS-PLA1 was observed in HCC patients compared to healthy control and correlated with PS-PLA1 mRNA in non-HCC tissues and with serum AST or ALT. Additionally, serum levels of PS-PLA1 were higher in HCC patients with HCV-related liver injury than in those with HBV or non-HBV-, non-HCV-related liver diseases. In conclusion, among LysoPS-related enzymes and receptors, PS-PLA1 and LPS1 mRNA were increased in HCC. Based on the correlation between the serum PS-PLA1 and the mRNA level of PS-PLA1 in non-HCC tissues, the liver may be the main source of serum PS-PLA1, and serum PS-PLA1 levels may be a useful marker for liver injury.

## Introduction

Over the last two decades, lysophospholipids (LPLs) have been studied widely as biologically active molecules that play multiple roles in carcinogenesis, neurogenesis, vascular development and immunity^1–4^. Among LPLs, the crucial roles of lysophosphatidic acid (LPA) and sphingosine 1-phosphate (S1P) have been extensively unveiled, exerting multiple cellular responses with G-protein coupled receptors (LPA1–6 and S1P1–5). We recently reported the involvement of LPA and S1P in the pathogenesis of hepatocellular carcinoma (HCC) and colon cancer^5–7^. On the other hand, the evidence has been relatively scarce showing the actions and the clinical roles of other LPLs, such as lysophosphatidylglycerol, lysophosphatidylserine (LysoPS), lysophosphatidylinositol, lysophosphatidylethanolamine or lysophosphatidylcholine.

Among these LPLs, we have been paying attention to LysoPS, the binding receptors of which were recently identified as LPS1 (GPR34), LPS2 (P2Y10) and LPS3 (GPR174) in humans and LPS2_L_ (A630033H20 or LPS2-like receptor) in rodents^8,9^. LysoPS is known to affect several cellular responses, including mast cell degranulation^10,11^, lymphocyte proliferation^12^, fibroblast migration^13,14^ and neurite outgrowth^15^. Regarding its receptors, LPS1 is expressed in many tissues, mostly in mast cells^16^ but it is not involved in the mast cell degranulation induced by LysoPS^17^. The roles of LPS2 and LPS3 remain to be elucidated at present, but the expressions of which were reported in lymphoid organs for LPS2, LPS3 and in the spleen and thymus for LPS2^18,19^. Among LysoPS receptors (LPSRs), the ectopic expression of LPS1 was reported in several solid tumors, such as stomach cancer^20^, with a potential correlation with clinico-pathological characteristics, tumor differentiation or prognosis. LysoPS stimulates the migratory ability of colon cancer cell lines through the LPS1 and PI3K/Akt pathways^21^.

Phosphatidylserine specific-phospholipase A1 (PS-PLA1), one of the enzymes that is responsible for LysoPS production and acts on PS in the cell bilayer, has the roles in the oncogenesis and metastasis of colorectal cancer in humans^22^. We have found evidence suggesting that the PS-PLA1-mediated pathway might be involved in the production of LysoPS in stomach cancer^23^. Furthermore, we have reported that the serum levels of PS-PLA1 are higher in melanoma subjects and associated with its clinical stages^24^. Because these lines of evidence may suggest a role for LysoPS in cancer, we sought to examine the potential involvement of LysoPS in the pathophysiology of HCC.

## Results

### mRNA levels of LysoPS-related enzymes and receptors in HCC

A total of 97 patients with HCC who underwent surgical treatment were analyzed, among whom, 61 were diagnosed with primary HCC and 36 with recurrent HCC. It is well known that HCC recurrence is frequently ectopic because the underlying chronic liver diseases continue to increase the risk for hepatocarcinogenesis in patients^25^. The clinical and laboratory characteristics of the patients are shown in Table 1.

**Table 1:** Patients’ characteristics.

Parameter	n = 97
Female/Male	20/77
Age (years)	69.8 (64.7–74.9)
BMI (kg/m^2^)	22.9 (21.1–25.4)
**Types of hepatitis**	
Hepatitis B (%)	18 (18.6)
Hepatitis C (%)	37 (38.1)
Alcoholic (%)	11 (11.3)
Others (%)	31 (31.9)
Patients with primary HCC/recurrent HCC	61/36
Maximum tumor diameter (cm)	2.8 (1.8–5.0)
**Number of tumors**	
Single (%)	65 (67.0)
More than 2 (%)	32 (33.0)
White blood cell count (×10^3^/μL)	5.30 (4.40–6.20)
Hemoglobin content (g/dL)	13.5 (12.3–14.3)
Platelet count (×10^4^/μL)	16.0 (12.6–19.4)
CRP (mg/dL)	0.07 (0.04–0.18)
Albumin (g/dL)	4.0 (3.7–4.3)
AST (U/L)	31.0 (25.0–54.0)
ALT (U/L)	29.0 (19.0–44.0)
GGT (U/L)	48.0 (30.0–97.0)
Total bilirubin (mg/dL)	0.7 (0.6–1.0)
Creatinine (mg/dL)	0.81 (0.66–0.98)
Triglyceride (mg/dL)	103 (78–134)
Total cholesterol (mg/dL)	174 (154–197)
Fasting blood glucose (mg/dL)	101 (93–125)
HbA1c (NGSP) (%)	5.9 (5.6–6.8)
PT-INR	0.96 (0.93–1.01)
ICGR15 (%)	11.5 (7.8–15.9)
AFP (ng/mL)	10.9 (4.1–88.5)
AFP-L3 (%)	3.0 (0.5–15.8)
DCP (mAu/mL)	31 (17.0–383.5)
**Background liver**	
Fibrosis stage 0/1/2/3/4	2/22/18/24/31
Activity grade 0/1/2	11/66/20
Tumor differentiation	
Well (%)	11 (11.3)
Well to moderate (%)	30 (30.9)
Moderate (%)	38 (39.2)
Moderate to poor (%)	14 (14.4)
Poor (%)	4 (4.1)
Microvascular invasion (+)/(−)	22/75

Spearman’s rank correlation was used to test the associations.

**Figure 1:**
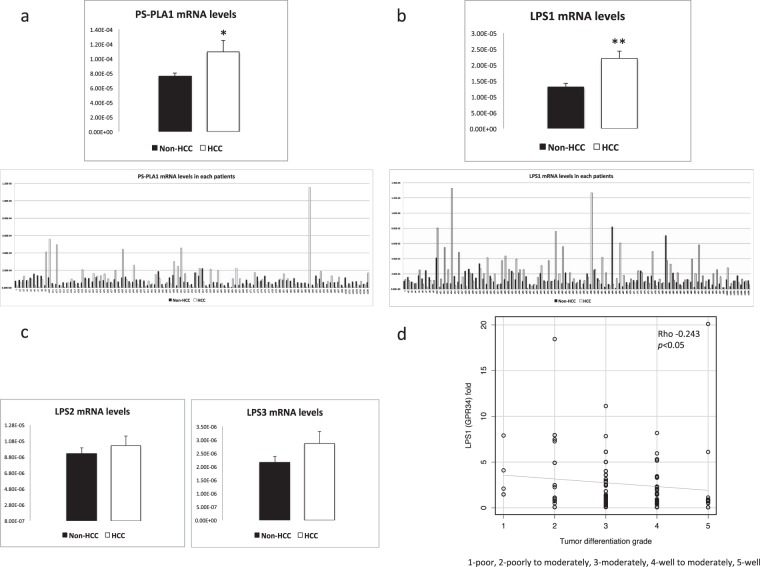
mRNA levels of LysoPS-generating enzyme and its receptors. (**a**) PS-PLA1 mRNA levels were increased (*p < 0.05) in HCC tissues in comparison to non-HCC tissues in 42 of 97 patients, being 1.4-fold higher in HCC tissues than non-HCC tissues. (**b)** Higher mRNA levels of LPS1 in HCC tissues were observed in 61 of 97 patients (**p < 0.01), being 1.7-fold higher in HCC tissues than non-HCC tissues. (**c**) LPS2, LPS3 mRNA levels did not differ between HCC tissues and non-HCC tissues. (**d**) LPS1 mRNA levels in HCC were correlated with tumor differentiation.

We first examined the mRNA levels of LysoPS-related enzymes and receptors in HCC tissues in comparison with adjacent non-HCC tissues. As depicted in Fig. 1a,b increased mRNA levels in HCC compared to non-HCC tissues were observed for PS-PLA1 (P < 0.05) and LPS1 (P < 0.01). Higher mRNA levels of PS-PLA1 and LPS1 in HCC tissues were observed in 42 of 97 (43.3%) and 61 of 97 patients (62.8%), respectively. The mean mRNA levels of PS-PLA1 and LPS1 were 1.4- and 1.7-fold higher, respectively, in HCC tissues than in non-HCC tissues (Fig. 1a,b). In contrast, no differences were observed between HCC tissues and non-HCC tissues regarding the mRNA levels of the other two LysoPS receptors, LPS2 (P = 0.57) and LPS3 (P = 0.16) (Fig. 1c).

To explore the features of HCC with enhanced mRNA levels of PS-PLA1 or LPS1, potential relationships with clinical parameters were analyzed, where there were no correlation between mRNA levels of PS-PLA1 in HCC and the clinical parameters. On the other hand, the increase in mRNA levels of LPS1 in HCC tissues, expressed as fold, was correlated with tumor differentiation; the differentiation levels of HCC were poorer with higher mRNA levels of LPS1 (Fig. 1d); however, prognosis was not different according to mRNA levels of LPS1.

### Serum levels of PS-PLA1 in HCC patients

**Figure 2:**
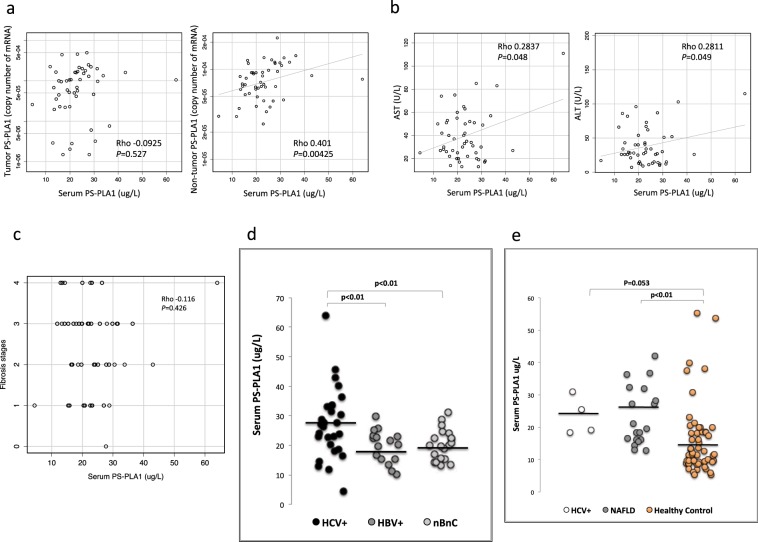
Serum PS-PLA1 levels and their correlation with various parameters. (**a**) Serum PS-PLA1 levels were correlated with the mRNA levels of PS-PLA1 in non-HCC tissues but not in HCC tissues. (**b)** The relationship between serum PS-PLA1 levels and clinical markers. A strong correlation was observed with serum levels of AST and ALT (**b**) but not with background liver fibrosis stages (**c**). (**d)** Serum PS-PLA1 levels in HCC patients with different etiologies. Serum PS-PLA1 levels in HCC patients with HCV infection were compared to those with HBV infection or non-HBV, non-HCV-related liver disease. (**e)** Serum PS-PLA1 levels in liver injuries without HCC. Serum PS-PLA1 levels were increased in non-HCC patients with liver cirrhosis caused by HCV (23.5 ± 5.9 μg/L) or non-alcoholic fatty liver disease (23.4 ± 9.1 μg/L) in comparison to healthy subjects (15.4 ± 10.7 μg/L).

In these patients with HCC, serum levels of PS-PLA1 were measured as 23.3 ± 9.4 μg/L, which were significantly higher than those in 59 healthy subjects, 15.4 ± 10.7 μg/L, reported in our previous study^24^ (P < 0.01). Although increased mRNA levels in HCC tissues may be responsible for the increase in serum PS-PLA1 levels, at least in part, serum PS-PLA1 levels were not correlated with PS-PLA1 mRNA levels in HCC tissues but were correlated with those in non-HCC, background tissues (Fig. 2a). Furthermore, serum PS-PLA1 levels were not correlated with any parameters for HCC, including its size or HCC markers, such as AFP or PIVKA II (Table 2). Among the parameters in general, serum PS-PLA1 levels were strongly correlated with serum levels of AST and ALT (Fig. 2b, Table 2) but not with platelet counts and serum albumin levels (Table 2). Histological findings consistently revealed no association between the serum PS-PLA1 levels and the fibrosis stages of non-HCC, background tissues (Fig. 2c).

**Table 2:** Relationships between serum PS-PLA1 and parameters for HCC.

Parameter	Serum PS-PLA1	
	Spearman’s rho	*P* value
Tumor size (cm)	0.099	0.946
Number of tumors	−0.0241	0.86
Degree of tumor differentiation	0.049	0.7373
AFP (ng/mL)	0.0888	0.52
AFP-L3 (%)	−0.0181	0.218
PIVKA-II (mAu/mL)	−0.0752	0.632
AST (U/L)	0.2837	0.048
ALT (U/L)	0.2811	0.049
Platelet counts	−0.1727	0.2353
Albumin	−0.1799	0.216

Spearman’s rank correlation was used to test the associations.

Next, we examined serum PS-PLA1 levels in HCC patients according to the background liver diseases. Figure 2d demonstrates that serum PS-PLA1 levels were higher in HCC patients with hepatitis C-related chronic liver disease than those with hepatitis B-related chronic liver disease or those with non-HBV, non-HCV-related chronic liver disease. The current results suggest that the background liver of HCC may be involved in the regulation of serum PS-PLA1 levels as the site of production. Thus, we further wondered whether serum PS-PLA1 levels might be increased in liver injury without HCC. Indeed, serum PS-PLA1 levels were higher in patients with non-alcoholic fatty liver disease (NAFLD), 23.4 ± 9.1 μg/L (P < 0.01, n = 19), compared to those in healthy subjects (Fig. 2e), and tended to be higher in patients with liver cirrhosis caused by hepatitis C virus, 23.5 ± 5.9 μg/L, though not significant due to the small sample size (P = 0.053, n = 4). Although the exact mechanism(s) should be further elucidated, the high levels of serum PS-PLA1 in non-alcoholic fatty liver disease are noted.

## Discussion

In the current study, the increased mRNA levels of a generating enzyme, PS-PLA1, and a receptor (LPS1) of LysoPS were observed in HCC tissues compared to non-HCC tissues. We then examined the potential relationships between the mRNA levels of PS-PLA1 or LPS1 in HCC and its clinical parameters. We found only a correlation between higher LPS1 mRNA levels in HCC and poorer degree of differentiation, but no other correlations between PS-PLA1 mRNA levels and the clinical parameters were observed. However, the prognosis of HCC was not different according to LPS1 mRNA levels. This is because the curative operation was performed in the patients enrolled, obscuring the effects of tumor differentiation and hence LPS1 on prognosis.

In rodents, PS-PLA1 is reportedly released from activated platelets^26^, but regarding humans, its release is to be elucidated. The current study suggests that the liver may play a role in the regulation of serum PS-PLA1 levels, based on the evidence that serum PS-PLA1 levels were correlated with mRNA levels of PS-PLA1 in non-HCC, background liver tissue but not with those in HCC tissue. We would assume that because the size of HCC treated with surgical resection in the current study was relatively small, enhanced PS-PLA1 production in HCC might not affect serum PS-PLA1 levels. It should be clarified whether serum PS-PLA1 levels might be useful as a marker of HCC with larger sized tumors. Nonetheless, in line with the current finding, serum PS-PLA1 levels varied according to the etiology of the background liver tissue, and serum PS-PLA1 levels were increased in patients with liver injury in the absence of HCC. Because serum PS-PLA1 levels were correlated with serum levels of AST and ALT, it is speculated that hepatocyte injury may cause active production of PS-PLA1 in the liver and hence may lead to an increase in serum PS-PLA1 levels.

As described in the introduction, the evidence showing the clinical significance of PS-PLA1 has been very scarce. As a limitation of our current study, we could just find the regulatory mechanism of serum PS-PLA1 levels, at least in part, without clarifying the clinical significance of PS-PLA1. In the current study, we observed elevated serum PS-PLA1 in patients with NAFLD, which suggests the involvement of the PS-PLA1 in the metabolic syndrome. Similar to PS-PLA1, upregulation of LPS1 in HCC has been unveiled in the current study, however, its significance remains obscure. Although we could find the supportive data for a link between HCC and PS-PLA1 or LPS1, the data are phenomenological at present and should be elucidated further.

## Patients and Methods

### Patients

Patients with HCC who were treated at the Hepatobiliary Pancreatic Surgery Division, Department of Surgery, the University of Tokyo Hospital, between January 2013 and June 2015 provided consent to be enrolled in this study. All of the enrolled patients underwent liver resection, among whom, 61 patients developed primary HCC and 36 had recurrent HCC, from whom sufficient HCC tissues and adjacent non-tumorous tissues for the analyses were obtained. None of the patients received pre-op treatment.

This study was conducted in accordance with the ethical guidelines of the 1975 Declaration of Helsinki and was approved by the Institutional Research Ethics Committee of the University of Tokyo. Written informed consent was obtained from the patients for the use of clinical samples.

### Quantitative real-time PCR of LPSRs and PS-PLA1

Quantitative real-time PCR was performed as previously described^6^. Human LPS1, LPS2, LPS3, PS-PLA1 and eukaryotic ribosomal 18 S primers and probes (TaqMan Gene Expression Assays) were obtained from Applied Biosystems (Hs00271105_s1, Hs00274326_s1, Hs00261404_s1, Hs01056915_m1 and Hs99999901_s1). The reaction conditions were as follows: incubation for 10 min at 95 °C, followed by 40 cycles at 95 °C for 15 sec and 60 °C for 1 min. The target gene mRNA expression levels were quantified relative to the 18S ribosomal RNA using the 2^−ΔΔCt^ method (Applied Biosystems, User Bulletin No. 2).

### Measurement of serum PS-PLA levels

Serum samples were collected after patient admission and before surgical procedure. Serum levels of PS-PLA1 were measured using a two-site immunoenzymatic assay in the TOSOH AIA system (TOSOH, Tokyo, Japan)^27^.

### Statistical analysis

All tests for significance were two-tailed, and P < 0.05 was considered statistically significant. The cumulative incidence of intra and extrahepatic recurrence was calculated using the Kaplan-Meier method, and differences among groups were assessed using the log-rank test. A paired t-test was used to analyze differences in mRNAs levels of LPSRs in tumors and corresponding nontumorous tissues. Data processing and analysis were performed using R statistic software version 3.3.1 (http://www.r-project.org).

The results of the *in vitro* experiments are expressed as the means and standard deviations (SDs). The results were considered significant when P-values were < 0.05.
